# Assessing the impact of parents’ digital and health literacy on children’s participation in sport

**DOI:** 10.1093/heapro/daaf038

**Published:** 2025-04-23

**Authors:** Ayşe Gül Güven, Yasemin Nuran Dönmez, Fatma İncedere, Medine Ayşin Taşar

**Affiliations:** Division of Adolescent Medicine, Department of Pediatrics, Ankara Training and Research Hospital, University of Health Sciences, Hacettepe Mh.Ulucanlar Cd.No:89, Altındağ/Ankara, 06230 Ankara, Turkey; Division of Adolescent Medicine, Department of Pediatrics, Faculty of Medicine, University of Ankara, Balkiraz Mahallesi, Tıp Fakültesi Cd. No: 1/4, Mamak/Ankara, 06590 Ankara, Turkey; Department of Pediatric Cardiology, Ankara Training and Research Hospital, University of Health Sciences, Hacettepe Mh. Ulucanlar Cd.No: 89, Altındağ/Ankara, 06230 Ankara, Turkey; Department of Pediatric Cardiology, Ankara Training and Research Hospital, University of Health Sciences, Hacettepe Mh. Ulucanlar Cd.No: 89, Altındağ/Ankara, 06230 Ankara, Turkey; Department of Pediatrics, Ankara Training and Research Hospital, University of Health Sciences, Hacettepe Mh. Ulucanlar Cd. No:89, Altındağ/Ankara, 06230 Ankara, Turkey

**Keywords:** digital technology, health literacy, sports, parents, children

## Abstract

Higher levels of parental health and digital literacy are associated with better health knowledge and therefore better health outcomes for their children. There is currently no research evaluating the impact of parental digital and health literacy on children’s participation in sport. This cross-sectional study aimed to investigate the effect of parental digital and health literacy on the sport participation of their children and included parents of children aged 6–18 years, categorized into a sport-participating group (*n* = 201) and a non-participating group (*n* = 116). Parents completed a questionnaire assessing demographic characteristics, internet and mobile phone usage for health-related purposes, and their children’s level of sport participation. Additionally, they were administered the Digital Literacy Scale and the Health Literacy Scale. The total, technical, and social dimension scores of the Digital Literacy Scale were significantly higher in parents of children participating in sport (*P* < .05). Similarly, the total score on the Health Literacy Scale, as well as the subscale scores for accessing/obtaining, understanding, and processing/appraising health-related information were significantly higher in the sport-participating group (*P* < .05). Furthermore, a significant correlation was observed between the total scores of the Digital Literacy Scale and the Health Literacy Scale (*P* = .001, *r* = 0.412). These findings indicate that parents of children engaged in sport have significantly higher digital and health literacy levels. Enhancing parental digital and health literacy may play a crucial role in promoting children’s participation in sport. Interventions aimed at improving parental digital and health literacy could positively impact children’s sport-related health outcomes.

**Contribution to Health Promotion**

- This study aims to evaluate the influence of parental digital and health literacy on children’s levels of physical activity.
- The findings highlight the role of parents’ ability to navigate digital platforms and access health-related information in guiding their children’s engagement in physical activity.
- Improving parents’ knowledge and skills in digital and health literacy can help them to better encourage and support their children’s participation in sport.

## INTRODUCTION

Participation in sport is widely recognized as an important contributor to the development of psychosocial benefits in children, including increased self-confidence, enhanced academic performance, higher perceived self-esteem, and improved emotional regulation (Holt et al. 2011, Neely and Holt 2014). In addition to its developmental and psychosocial effects, sport also minimizes health-related conditions such as obesity (Velardo et al. 2010). As parents have a significant influence on children’s participation in sport, parents’ perceptions of the benefits of sport participation have a major impact on a child’s regular participation in sport (Velardo et al. 2010). Families’ approach to children’s participation in sport, whether as supporters, guides or role models, can significantly influence children’s self-perceptions, thoughts, and motivations about their abilities, either positively or negatively (Neely and Holt 2014). The family’s level of education, income, marital status, and occupation has a significant impact on their perspective of taking a supportive role in their child’s participation in sport (Velardo et al. 2010). Participation in sport tends to be higher among those from higher-income families and lower among those from lower-income families (Holt et al. 2011). Strong and supportive family interaction plays a crucial role in promoting sport participation.

Health literacy is defined as ‘the knowledge, motivation and skills related to reading, writing and speaking that enable people to access, understand, evaluate and use the health information they need to make decisions about their health in their daily lives, to improve their health and to prevent illness in order to improve and maintain their quality of life’ (Sørensen et al. 2012). Parents with a sufficient level of health literacy ought to be equipped to take responsibility not only for maintaining their own health but also for ensuring the well-being of their children (Sørensen et al. 2012). While high parental health literacy is associated with positive health behaviours such as increased physical activity, low parental health literacy has been identified as a contributing factor to childhood obesity (Nakajima et al. 2024).

Health literacy is shaped by a variety of factors, including an individual’s health behaviours, their level of health knowledge, and the amount of social support they receive (Straughan and Xu 2023). These elements work together to determine how effectively a parent can access, understand, and use health information to make informed decisions for their children. Families are required to improve their understanding, motivation, and awareness of novel health-related concepts and situations, integrating them into their daily lives to prevent disease and enhance life quality (Sørensen et al. 2012). Low health literacy among parents is associated with challenges in following instructions, applying health knowledge in practice, analysing health information, and coping with health-related problems (Jacobsson et al. 2021). Improving health literacy equips children to better manage both health and psychosocial challenges in the future, supporting their long-term participation in sport (Jacobsson et al. 2021).

Although the definition of digital literacy is still being debated, according to the American Library Association’s Digital Literacy Task Force, a digitally literate person has the skills (technical and cognitive) necessary to locate, understand, evaluate, create, and communicate digital information in a variety of formats (Fulton and McGuinness 2016). He/she is able to use a variety of technologies appropriately and effectively to retrieve information, interpret results, and evaluate the quality of that information; and has an understanding of technology, lifelong learning, and life-wide learning; understands the relationship between personal privacy and information management; uses these skills and appropriate technologies to communicate and collaborate with peers, colleagues, family, and sometimes the public; and uses these skills to actively participate in civil society and contribute to an informed and engaged community (Fulton and McGuinness 2016). In short, digital literacy is the combination of attitudes, knowledge, and skills needed to live, learn, and work in a digital society (JISC 2022).

In the digital age, parents with digital literacy skills can act as important supporters for their children. Digital literacy requires a combination of intellectual and technical skills (Kim et al. 2022). By recognizing the potential of the internet, electronic sources, or digital tools (performance tracking devices, training and exercise apps, injury prevention devices, and wearable sports technology), they are able to access, evaluate, and share information through digital technologies (Smith and Magnani 2019, Li et al. 2022, Yuen et al. 2024). Parents’ parenting styles can be influenced by their experiences with technology, leading to changes in their skills and attitudes (Terras and Ramsay 2016). Families have a responsibility to learn and explore sports-related information with their children. This helps to improve their children’s skills and performance, while also instilling ethical values (Li et al. 2022). These skills enable parents to guide their children in navigating the digital world responsibly, fostering informed decision-making and critical thinking in the process (Yuen et al. 2024). In addition, awareness of digital platforms, sensors, virtual training, wearable sports technologies, and digital protection tools allows families to support training and help prevent injury (Hertling et al. 2022, Li et al. 2022). Digital health literacy, like health literacy, is influenced by various factors such as education level, occupation, and income (Yuen *et al*. 2024). Limited digital health literacy can lead to reduced confidence in evaluating digital health information. Factors such as low socioeconomic status, limited access to digital tools and minimal use are often associated with low digital health literacy. On the other hand, higher levels of digital health literacy are associated with positive health outcomes (Yuen et al. 2024).

Despite some research on assessing digital and health literacy among parents of children and adolescents, there is a research gap in understanding the importance of these skills among parents of children participating in sport. Assessing health and digital literacy, improving the literacy levels of low-literate parents, and identifying the impact of literacy levels on sport participation and health outcomes are valuable initiatives. There is a need for more qualitative studies to understand family motivation and support for children’s sport participation. Understanding the barriers will encourage children to engage in sport both emotionally and cognitively, helping them to develop physical activity habits and contributing to their social and behavioural development. The primary aim of this study was to compare the levels of health literacy and digital literacy among parents of children who participate in sport with those who do not. This research is interested in investigating the influence of parents’ knowledge and use of health-related and digital resources on their ability to encourage and motivate their child’s participation in sport.

## MATERIAL AND METHODS

### Participants

This cross-sectional study was conducted by the Adolescent Medicine, General Pediatrics and Pediatric Cardiology Department of the University of Health Sciences Ankara Training and Research Hospital between December 2023 and May 2024. Parents whose children participated in sport between the ages of 6 and 18 (*n* = 201) and those who did not participate in sport (*n* = 116) were included in the study. The sport participant group consisted of children who participated in sports, whether casually, in a club, competitively or just for fun, and applied for a sports licence at the Pediatric Cardiology Department, and the control group consisted of children who did not participate in sports, had no underlying chronic disease or complaint, and applied to the General Pediatrics and Adolescent Medicine Department for growth and development monitoring. After obtaining consent, parents of children attending these clinics were given a general information form as well as a Health Literacy and Digital Literacy Scale. The surveys were self-administered and a pilot test was conducted with a small number of parents (*n* = 15) before data collection began to gather input on item comprehension and time taken to complete the form.

### Study protocol

#### Socio-demographic information questions

The questions were designed by the authors according to literature and concerned the age and the gender of the parents/children, the marital status of the parents (married, single), the educational level of the parents (primary school, high school, and ≥ university), the employment status of the mother (housewife, working), the employment status of the father (unemployed, officer, self-employed), the family income [low (income less than expenditure), medium (income equals expenditure), high (income higher than expenditure)], the number of people living at home, the presence of chronic diseases in the parents, the presence of chronic diseases in the relatives in the family, and continuous use of medication by parents or relatives.

#### Questions on parents’ internet and mobile phone use for health management

Questions designed by the authors were asked about whether the parent uses a mobile phone and the National Personal Health System application (a mobile application linked to the Ministry of Health that contains personal health data), whether the parent searches the internet for diagnoses of illnesses diagnosed in themselves or in people around them, and whether the parent searches the internet for side effects of prescribed medicines.

#### Questions to determine the training intensity of children and the level of participation in competitions

Parents were asked questions about their children’s participation in competitions within the province and about the intensity of training. Weekly training hours and intensity for children who participated in sports were categorized as 1–2 h per day for less than 3 days per week (group 1), 1–2 h per day for 3–4 days per week (group 2), and 2 h or more per day for 5 or more days per week (group 3).

### Digital Literacy Scale

The Digital Literacy Scale was developed by Ng (2012) and the Turkish validity and reliability study was conducted by Hamutoğlu et al. (2017) (see Supplementary S1). It consists of 17 items in a five-point Likert style. All items of the scale are positive and are scored between 1 and 5. It has four sub-dimensions: attitude, technical, cognitive, and social. As there are seven items in the attitude sub-dimension, the lowest score that can be obtained from this dimension is 7 and the highest score is 35. There are six items in the technical dimension; the lowest score that can be obtained from this dimension is 6 and the highest score is 30. There are two items in the cognitive sub-dimension; the lowest score that can be obtained from this dimension is 2 and the highest score is 10. As there are two items in the social dimension, the lowest score that can be obtained from this dimension is 2 and the highest score is 10. Low scores indicate an inadequate/low level of digital literacy and high scores indicate a high level of digital literacy. Cronbach’s alpha coefficient for the sub-dimensions was found as 0.88 for attitude sub-dimension, 0.89 for technical sub-dimension, 0.70 for cognitive sub-dimension, and 0.72 for social sub-dimension. Cronbach’s alpha coefficient for the whole scale was found to be 0.93 (Hamutoğlu et al. 2017) (See Supplementary File S2).

### Health Literacy Scale

The Health Literacy Scale was developed by Toçi et al. (2015) and the Turkish validity and reliability of the scale was conducted by Temel and Aras (2017) (See Supplementary File S2). All structures of the scale are positive and consist of 25 items in a five-point Likert style and are scored between 1 and 5. The scale has four sub-dimensions: access/obtain information relevant to health, understand information relevant to health, process/appraise information relevant to health, and apply/use information relevant to health. Access/obtain information includes five items (1–5); the minimum score to be obtained from this subscale is 5 and the maximum score is 25. Understanding information contains seven items (items 6–12); the minimum score to be obtained from this subscale is 7 and the maximum score is 35. The process/appraise sub-dimension includes eight items (items 13–20); the minimum score to be obtained from this sub-dimension is 8 and the maximum score is 40. The apply/use sub-dimension also includes five items (items 21–25); the minimum score to be obtained from this sub-dimension is 5 and the maximum score is 25. The minimum score for the total scale is 25 and the maximum score is 125. Low scores indicate inadequate health literacy and high scores indicate adequate health literacy. The Cronbach’s alpha coefficient for the whole scale was found to be 0.92 and for the sub-dimensions access/ obtain information 0.71, understand, process, and appraise information 0.66 and apply/use 0.62 (Temel and Aras 2017).

### Ethical considerations and data collection

The survey protocol adhered to the ethical guidelines for medical and health research involving human subjects, was conducted in accordance with the declaration of Helsinki, and was approved by the Ethical Review Board of Ankara Training and Research Hospital (decision number: 1451). The survey was conducted only with respondents who gave informed consent. Respondents were informed that they could decide to participate or withdraw from the study at any time.

### Statistical analyses

Statistical analyses were performed with SPSS 26.0 for Windows. Descriptive values were expressed as frequency (*n*), percentage (%), mean, standard deviation (SD), and median. Categorical variables were compared using Pearson chi-squared tests, and continuous variables were compared using Student’s *t* and ANOVA tests if they followed a normal distribution according to the Kolmogorov–Smirnov and Shapiro–Wilk tests, and Mann–Whitney U and Kruskal–Wallis tests if they did not follow a normal distribution. The relationship between continuous variables was assessed using Spearman’s correlation analysis in cases of normal distribution and Pearson’s correlation analysis in cases of non-normal distribution. According to the correlation coefficient, the relationship status is 0–0.24 weak relationship, 0.25–0.49 moderate relationship, 0.50–0.74 strong relationship, and 0.75–1.00 very strong relationship. The effect of different independent variables on the child’s participation in sport was assessed using logistic regression analysis. Variables with *P* < .25 in the individual analyses were included as independent variables in the regression analysis. A *P* value < .05 was considered significant for all analyses.

## RESULTS

### Comparison of Digital and Health Literacy Scale scores of parents

The total score of the Digital Literacy Scale was found to be 3.69 ± 0.75 in the whole study group and was statistically higher in the sport-participating group than in the non-participating group (*P* = .014). The total score of the Health Literacy Scale was found to be 4.23 ± 0.64 in the whole study group and was statistically higher in the sport-participating group than in the non-participating group (*P* = .011) (Table 1). The comparison of total and sub-dimensional analyses of the Digital and Health Literacy Scale for parents is seen in Table 1.

**Table 1.** Comparison of total and sub-dimensional analyses of the Digital and Health Literacy Scale for parents of sport-participating and non-sport-participating group

	Sport-participating group (*n* = 201)	Non-sport-participating group (*n* = 116)	*P*
**Digital Literacy Scale**			
Attitude	3.75 ± 0.83	3.59 ± 0.87	0.115
Technical	3.82 ± 0.79	3.53 ± 0.96	**0.007**
Cognitive	3.83 ± 0.83	3.67 ± 1.0	0.145
Social	3.63 ± 0.88	3.38 ± 1.08	**0.029**
Total score	3.77 ± 0.71	3.56 ± 0.81	**0.014**
**Health Literacy Scale**			
Access/obtain information relevant to health	4.38 ± 0.69	4.03 ± 0.88	**0.001**
Understand information relevant to health	4.26 ± 0.76	4.05 ± 0.72	**0.011**
Process/appraise information relevant to health	4.31 ± 0.69	4.14 ± 0.81	**0.046**
Apply/use information relevant to health	4.34 ± 0.67	4.21 ± 0.79	0.119
Total score	4.32 ± 0.59	4.11 ± 0.68	**0.004**

Statistically significant variables are shown as bold.

According to weekly training hours, no significant difference was observed between the two groups in the total and sub-dimension scores of the Digital and Health Literacy Scale, except for the cognitive sub-dimension of the Digital Literacy Scale (*P* > .05). The cognitive sub-dimension scores of the parents in group 2 were higher than those in the other groups (*P* = .042).

There was also a correlation between the total score of the Digital and Health Literacy Scale (*P* = .001, *r* = 0.412).

### Characteristics of participants

The median age of the children of the 317 parents included in this study was found to be 12.8 years (range 6–18 years). The demographic characteristics of the study population are presented in Table 2. A significant difference was found between the parents of the sport-participating group and the non-participating group in terms of child gender, parental education level, parental employment status, and parental income level (*P* < .05) (Table 2).

**Table 2.** Comparison of the demographic characteristics of parents and children in the sport participating and non-participating group

	Sport participating group (*n* = 201)	Non-sport participating group (*n* = 116)	*P*
**Parent** (*n*,%)			0.247
Mother	152 (75.6)	95 (81.9)	
Father	49 (24.4)	21 (18.1)	
Age of the child (y)^*^	12.8 (6–18)	13.5 (6.0–17.9)	0.529
**Gender of child** (*n*,%)			**0.036**
Female	95 (47.2)	69 (59.5)	
Male	106 (52.8)	47 (40.5)	
**Parental age** (y)^*^	40 (26–60)	40.5 (23–54)	0.696
**Parents’ marital status** (*n*,%)			0.389
Married	184 (91.5)	110 (94.8)	
Single	17 (8.5)	6 (5.2)	
**Parents’ education level** (*n*,%)			**0.001**
Primary	66 (32.8)	60 (51.7)	
High school graduate	66 (32.8)	42 (36.2)	
≥University	96 (34.4)	14 (12.1)	
**Employment status of mother** (*n*,%)			**0.001**
Housewife	123 (61.2)	99 (85.3)	
Working mother	78 (38.8)	17 (14.7)	
**Employment status of father** (*n*,%)			**0.020**
Unemployment	13 (6.5)	13 (11.2)	
Officer	93 (46.3)	36 (31.0)	
Self-employed	95 (47.2)	67 (57.8)	
**Level of parental income** (*n*,%)			**0.007**
Low	51 (25.4)	25 (21.6)	
Middle	103 (51.2)	78 (67.2)	
High	47 (23.4)	13 (11.2)	
**Number of individuals living at home** ^*^ (*n*,%)	4 (1–8)	4 (1–10)	**0.004**
**Parents with a chronic illness** (*n*,%)			**0.012**
Yes	26 (12.9)	28 (24.1)	
No	175(87.1)	88(75.9)	
**Relatives with a chronic illness** (*n*,%)			**0.015**
Yes	79 (39.3)	62 (54.4)	
No	122 (60.7)	54 (46.6)	
**Chronic medication use by parents or relatives** (*n*,%)			**0.019**
Yes	92 (45.8)	69 (59.5)	
No	109 (54.2)	47 (40.5)	

Statistically significant variables are shown as bold.

^*^indicates median (minimum–maximum values).

When the relationship between weekly hours of training and the socio-demographic characteristics of the participants was evaluated, significant differences were observed between the groups in terms of the child’s age, the mother’s marital status, and the parent’s level of education (*P* < .05) (Table 3).

**Table 3.** Characteristics of the parents and children according to the number of hours of training per week of the children

Weekly training days	Group 1 (*n* = 87)	Group 2 (*n* = 89)	Group 3 (*n* = 25)	*P*
Age of the child (y)^*^	12 (6–17.1)	12.8 (6.2–18)	14.3 (9.9–18)	**0.001**
**Gender of child** (*n*,%)				0.542
Female	42 (48.3)	39 (43.8)	14 (56.0)	
Male	45 (51.7)	50 (56.2)	11 (44.0)	
**Parental age** (y)^*^	40 (26–58)	39 (27–60)	43 (33–154)	0.134
**Parents’ marital status** (*n*,%)				**0.010**
Married	82 (94.3)	83 (93.3)	19 (76.0)	
Single	5 (5.7)	6 (6.7)	6 (24.0)	
**Parents’ education level** (*n*,%)				**0.006**
Primary	24 (27.6)	38 (42.7)	4 (16.0)	
High school	27 (31.0)	24 (27.0)	15 (60.0)	
≥University	36 (41.4)	27 (30.3)	6 (24.0)	
**Employment status of mother** (*n*,%)				0.459
Housewife	52 (59.8)	58 (65.2)	13 (52.0)	
Working mother	35 (40.2)	31 (34.8)	12 (48.0)	
**Employment status of father** (*n*,%)				0.263
Unemployment	43 (49.4)	39 (43.8)	11 (44.0)	
Officer	42 (48.3)	42 (47.2)	11 (44.0)	
Self-employed	2 (2.3)	8 (9.0)	3 (12.0)	
**Level of family income** (*n*,%)				0.843
Low	43 (49.4)	49 (55.1)	11 (44.0)	
Middle	22 (25.3)	21 (23.6)	6 (24.0)	
High	22 (25.3)	19 (21.3)	8 (32.0)	
**Number of individuals living at home** ^*^ (*n*,%)	4 (2–8)	4 (1–6)	4 (2–7)	0.535

Statistically significant variables are shown as bold.

^*^indicates median (minimum–maximum values).

When examining the relationship between parental socio-demographic characteristics and total scores on the Digital/Health Literacy Scale, it was found that mean total scores on the health (*P* = .001) and digital (*P* = .005) literacy scales increased significantly with increasing levels of education. It was observed that the digital literacy total scores of married parents were significantly higher than those of single parents (*P* = .015), and the health literacy total scores of working mothers were significantly higher than those of housewives (*P* = .034). There was no association between total scale scores and other socio-demographic characteristics (*P* > .05).

A weak correlation (*P* = .033, *r* = −0.120) was observed between the Health Literacy Scale total scores and parental age. There was no correlation between total Digital Literacy Scale scores and parental age (*P* = .307, *r* = −0.058), children’s age (*P* = .947, *r* = 0.004), and number of people living at home (*P* = 0.585, *r* = −0.031).

### Parents’ internet and mobile phone usage data for health management

The frequency of mobile phone use in the whole study group (*n* = 313) was found to be 98.7%, and there was no difference between the group that participated in sport and the group that did not participate in sport in terms of parents’ mobile phone use (*P* = 1.000), using the national personal health system application (*P* = .052), searching the internet for diagnoses of illnesses that had been diagnosed for themselves or people in their life (*P* = .724), and searching the internet for side effects of their prescribed medication (*P* = 1.000).

There was also no relationship between the frequency of children’s training and parents’ use of mobile phones and the national personal health system application and searching the internet for diagnoses of illnesses that had been diagnosed for themselves or people in their lives, and searching the internet for side effects of their prescribed medication (all *P* > .05).

The results of the relationship between digital and health literacy total scores and parental use of the internet and mobile phones for health management are shown in Table 4. A significant relationship was observed between the parents’ digital literacy total scores and using the national health system application (*P* = .038), searching on the internet about the disease diagnoses of themselves and the people around them (*P* = .006), and looking at the side effects of the drugs prescribed to them (*P* = .003).

**Table 4.** Relationship between total Digital and Health Literacy Scale scores and parental use of internet and mobile phones for health management

	Digital Health Literacy Scale total score (mean ± SD)	*P*	Health Literacy Scale total score (mean ± SD)	*P*
Using a mobile phone		0.436		0.952
Yes	3.69 ± 0.76		4.24 ± 0.64	
No	4.62 ± 2.07		4.22 ± 0.63	
Using national personal health system application		**0.038**		**0.031**
Yes	3.73 ± 0.77		4.27 ± 0.62	
No	3.47 ± 0.83		4.04 ± 0.68	
Do you search the internet for diagnoses of illnesses that have been diagnosed for you or for people in your life?		**0.006**		**0.001**
Yes	3.79 ± 0.71		4.32 ± 0.54	
No	3.53 ± 0.89		4.08 ± 0.77	
Do you look up the side effects of your prescribed medication on the internet?		**0.003**		0.155
Yes	3.78 ± 0.79		4.27 ± 0.64	
No	3.48 ± 0.74		4.15 ± 0.62	

Statistically significant variables are shown as bold.

Table 5 shows the associations between descriptive characteristics and children’s participation in sport.

**Table 5.** Multivariable logistic regression analysis of associations between descriptive characteristics with children’s sport participation

	OR	95% CI		*P*
		Lower	Upper	
**Health Literacy Scale total score**	1.018	1.001	1.034	**0.035**
**Child’s gender**				
Male (ref.: female)	1.895	1.125	3.192	**0.016**
**Parents’ education level**				
High school graduate (ref.: primary)	1.669	0.925	3.010	0.089
≥University (ref.: primary)	2.640	1.156	6.029	**0.021**
**Employment status of mother**				
Working (ref.: Housewife)	2.357	1.138	4.880	**0.021**
**Level of family income**				
High (ref.: Middle)	2.298	1.047	5.045	**0.038**
Low (ref.: Middle)	2.439	1.287	4.622	**0.006**
**Number of individuals living at home**	0.785	0.615	1.002	0.052
**Parents with a chronic illness**				
No (ref.: Yes)	2.008	0.985	4.094	0.055
**Chronic medication use by parents or relatives** No (ref.: Yes)	2.050	1.174	3.580	**0.012**

ref.: reference, OR: odds ratio, CI: confidence interval, Hosmer and Lemeshow test, *P*: 0.153, the final model was achieved in step 4.

It was observed that an increase in the total score of the Health Literacy Scale, the child’s gender being male, the participating parent being a university graduate, the mother being employed, the parents having high and low income, and the use of chronic medication by the parent or relative significantly increased the child’s participation in sport (*P* < .05).

## DISCUSSION

This cross-sectional study aimed to assess the level of health and digital literacy of parents of children participating in sport and to identify socio-demographic factors that influence their digital and health literacy and their children’s participation in sport. A significant difference was observed between the parents of children participating in sport and the non-participating group for the total, technical, and social sub-dimension scores of the Digital Literacy Scale, which were higher in the group whose children participated in sport (*P* < .05). In addition, the total score and the sub-dimension scores for access/receive health-related information, understand health-related information, and process/appraise health-related information on the Health Literacy Scale were all significantly higher for parents of children who participated in sport than for parents of children who did not participate in sport (*P* < .05).

A study (Mutz and Albrecht 2017) of 80 boys and 70 girls aged 6–11 years from three socially and ethnically mixed primary schools in Göttingen, Germany, examined the relationship between parents’ social status and children’s physical activity and reported that World Health Organization recommended (Chaput *et al*. 2020) levels of physical activity were related to gender (males more active) rather than age and that parents’ socioeconomic status (defined as education and income level) significantly influenced children’s physical activity levels. Another study was designed to include a total of 686 students (365 girls, 321 boys) from randomly selected schools in Belgium, Greece, Hungary, the Netherlands, and Switzerland from three cities of low/medium/high urbanization selected from each country. The aim of the study was to objectively assess the levels of sedentary time, light, moderate, and vigorous physical activity among 10- to 12-year-old students and to examine differences in sedentary time and physical activity levels according to gender and country. It was reported that girls spent significantly more time sedentary and less time in all physical activity intensities than boys (Verloigne *et al*. 2012). Similarly, in our study, which included children who lived in the same city but participated in sport at an outpatient clinic with parents from different socioeconomic levels, we found that females participated significantly less in sport than males. Intervention programmes to promote physical activity and reduce sedentary behaviour should be given particular attention to girls.

Post et al. (2018) designed a study to describe the socioeconomic status, measured by household income and educational attainment, of 949 parents with children (10–18 years) participating in youth club sports teams in Wisconsin using a non-validated questionnaire. It was concluded that children of parents reporting higher total household income or educational levels started participating in organized sports at a younger age and participated in sports more months per year than children of parents with lower total household income or educational levels. In our study, parental socioeconomic status (education level, employment status, and income level) was higher in children/who participated in sport. The parents who participated in our survey were recruited through convenience sampling from a limited geographical area. It is possible that different results would be obtained if recruitment were carried out in a wider geographical area or at different club sports facilities.

Previous empirical research (HLS-EU Consortium 2012) and theoretical models (Sørensen et al. 2012) on health literacy have shown that health literacy is correlated with socioeconomic status. A study by de Buhr and Tannen (2020) aimed to better understand health literacy in Germany and to assess its impact on children’s health. A large cross-sectional survey was conducted in 28 public primary and secondary schools in two German Länder. A total of 4217 parents completed a short form of the European Health Literacy Survey and a child questionnaire to assess children’s health behaviours. It was found that the main determinants of high parental health literacy were high socioeconomic status and older parental age, but in the multivariate model, only socioeconomic status remained significant. It was also stated that higher parental health literacy was associated with positive health behaviours in children, such as more physical activity. Although our study did not include as broad a group of parents as this study, it was conducted in a hospital setting where parents from different social and economic backgrounds apply for their children. Similar to the study above, we found that overall and many health literacy subscale scores and parental socioeconomic status (education level, current employment status, and income level) were significantly higher in parents whose children participated in sport. We also found that overall health literacy scores were correlated with parental age. Raising the socioeconomic status of parents can increase their health literacy and support them in encouraging their children to engage in more sports.

Our study showed an association between parental health and digital literacy. Similarly, in a randomized controlled trial in three urban paediatric clinics, Meyers et al. (2020) aimed to investigate how parental health literacy affects the use of internet and mobile phone technologies for health management. They found that parents with good health literacy used the internet and mobile phones more often and primarily for health management. Also, Pehora et al. (2015) reported that almost all parents used the internet to find health information for their children. It can be concluded that parents’ high rates of internet and mobile phone use to seek health care information related to their children as well as their willingness to use these methods to communicate with healthcare providers, demonstrate the promise of technology-based interventions to improve child health outcomes by facilitating access to and management of child health data.

In the systematic review, Mörelius et al. (2021) investigated the effect of digital health interventions on the health literacy of parents of children with chronic health problems, and all studies reported high satisfaction with the digital intervention. They also found that all studies reported improvements in parental health literacy after the intervention, either as increases in disease-specific knowledge or changes in health behaviour. More research should be done on these interventions, as increasing parents’ digital literacy may improve their access to and participation in these interventions, and therefore their children’s health.

In another review that looked at socio-demographic markers of digital health literacy, Estrela et al. (2023) stated that more research is needed. They also reported that age had a negative effect on the level of digital health literacy in the general population, gender had no effect, and education level and income level had a positive effect. In our study, we observed a weak negative correlation between parental age and health literacy total scores, while we did not observe a correlation with Digital Literacy Scale total scores. In the review described above, heterogeneity and diversity in the included study populations were given as limitations and it was stated that there may be a risk of participant inclusion bias since it was performed in selected populations, and in conclusion, it was stated that age is not a strong determinant of digital health literacy with current data.

### Limitations

One limitation is that the studies that have assessed digital literacy and health literacy are different from the scales that we have used in our study. Also, the scales we used to assess digital and health literacy in our study were structured as self-reports. The socioeconomic status variables were also self-reported and categorized, which limits the amount of information available and increases the risk of recall or social desirability bias. Future research should aim to use more reliable measures of socioeconomic status. It is a cross-sectional study, so the sample was not representative of the country as a whole and causal relationship could not be established. To address this limitation, future trials should use a longitudinal multicentre design. No distinction was made according to the participants’ preferred sports, as it may not be possible to reach a sufficient number of athletes in different sports groups. The effect of parental demographics on physical activity levels in children and adolescents may differ in studies with larger numbers of participants.

### Strengths of the study

Our study is the first to assess the digital and health literacy of parents of children who participate in sport. It is also the first study to examine the relationship between parents’ digital/health literacy and sport participation, one of the most important health-promoting and sustainable positive behaviours in childhood and socio-demographic data.

## CONCLUSION

In this study, the digital and health literacy scores of parents of children who participated in sport were higher than those of parents of children who did not participate in sport in univariate analysis. However, in regression analyses, only the total health literacy score of the parent was found to have an effect on the child’s participation in sport. Overall scores on the health and digital literacy scales increased significantly with increasing levels of parental education in univariate analysis. Based on our initial and preliminary findings, we believe that increasing levels of parental education will lead to important health outcomes, such as increasing digital and health literacy and engaging children in sport. Further studies with wider participation are needed to further elucidate the determinants of parental digital and health literacy.

## Supplementary Material

daaf038_suppl_Supplementary_Material

## Data Availability

The data that support the findings of this study are available from the corresponding author upon reasonable request.
