# Supplementary material for: Assessing the impact of parents’ digital and health literacy on children’s participation in sport
Source: Health Promot Int. 2025 Apr 23;40(2):daaf038. doi: 10.1093/heapro/daaf038 (PMC12015608; doi:10.1093/heapro/daaf038)
Supplement: daaf038_suppl_Supplementary_Material [file daaf038_suppl_supplementary_material.docx]

**Supplement 1; link:**

**Link:** <https://dergipark.org.tr/tr/pub/egeefd/issue/28714/295306>

**Supplement 2; link**

**Link:**<https://www.fnjn.org/en/evaluation-of-validity-and-reliability-of-the-turkish-version-of-health-literacy-scale-16690>
